# Analysis of Carbon Formation on Machined Leather Specimen Using FTIR Technique in Laser Diode Assisted Cutting Process

**DOI:** 10.3390/ma16010148

**Published:** 2022-12-23

**Authors:** Vasanth Swaminathan, Muthuramalingam Thangaraj, Edwin George Joseph, Suhaib Sulfikar Khadar, Joel Philip Saji, Panagiotis Karmiris-Obratański

**Affiliations:** 1 Department of Mechatronics Engineering, SRM Institute of Science and Technology, SRM Nagar, Kattankulathur 603203, Tamil Nadu, India; 2 Department of Mechanical Engineering, University of California, Berkeley, CA 94720-1740, USA; 3 School of Engineering and Physical Sciences, Heriot-Watt University, Edinburgh EH14 4AS, UK; 4 Department of Manufacturing Systems, Faculty of Mechanical Engineering and Robotics, AGH University of Science and Technology, 30-059 Cracow, Poland

**Keywords:** FTIR, carbonization, leather, laser, power diode

## Abstract

The leather materials are used in a multitude of sectors, including footwear, apparel, handicrafts, and the automotive industry. Due to the radiant heat generated by a laser beam, the laser cutting of leather results in a carbonized cut edge. There is currently no technology available for measuring the carbonization along the contour edges of leather. The purpose of this experimental investigation was to determine the impact of power diode-based laser cutting on the carbonization of machined buffalo leather with the help of a digital microscope to improve the machining process. The ATR-FTIR spectrum was used to analyze the carbon-related functional group in the mid-IR spectrum of carbonized leather samples. It was found that the proposed method can measure the amount of carbon deposition in the cutting zone. The lower amplitude duty cycle with higher feed rate can reduce carbon formation owing to the lower thermal energy distribution. The amplitude (4.5 V), duty cycle (70%) and feed rate (90 mm/s) can produce optimal performance measures.

## 1. Introduction

Leather is a material that has great commercial demand, as many products, including gloves, footwear, fashion and luxury automobile interiors are manufactured out of it. Cutting leather using a blade yields the greatest results from a structural standpoint. Die cutting (punching with a sharp-edged die) alters the structure of leather, which manifests as a jamming of the top layer of leather. Laser cutting induces carbonization on the machined edge due to a heat effect. This effect is especially noticeable in light-colored leathers [1]. The process is precise and does not cause the cloth to stretch as hand cutting would. Buffalo leather, which is commonly used to manufacture accessories, as well as clothing, was used as a specimen [2]. Due to the heat effect of the laser beam, cutting leather with a laser produces a carbonized cut edge. This is not the case with mechanical cutting techniques. Due to the thermal effect, laser cutting creates a minor carbonization of the cut edge. This look is particularly noticeable on lighter leather colors, such as white or yellow. This impact must be taken into account when considering laser cutting. Laser cutting leaves very imperceptible markings on leather, and the procedure gives the material a completed appearance. Comparing the edges of laser-cut and mechanically cut leather indicated that laser cutting induces carbonization of the cut edge, which was the most notable distinction between cutting methods. Depending on the color of the leather, the thermal impact of the laser beam on the edge of the cut varied. Laser-cut edges of dark, and especially brown leather, are deemed ideal for laser cutting, although laser-cut edges of light-colored leathers are immediately discernible. In general, laser cutting is the most suited method for cutting complicated geometries because of its great flexibility, ease of setup and nesting, rapid geometry changes, and excellent adaptability to diverse material characteristics (such as thickness). These characteristics make laser an interesting design tool. In contrast, the great flexibility, ease of setup and nesting, as well as the rapid cutting rates and consistent cutting quality, suggest industrial applications. The simple applicability of nesting and cutting sequence software in conjunction with laser technology is an additional factor indicating possible industrial uses. Carbonized cut edge may, however, be a limiting issue in this instance. After laser cutting, the removal of carbonized markings and smoke odor necessitates particular procedures that are unsuitable for mass manufacturing. In conclusion, artworks, design, and prototypes are the areas where laser technology’s advantages may be completely leveraged, while downsides can be avoided with very easy steps. A digital microscope was used to capture the contour edges of leather samples with high resolution. As a result it was observed that the buffalo skin had minute wavy pores [3]. In view of this, a scanning electron microscope (SEM)-based visual analysis of leather was developed [4]. The objective of this research is to analyze and quantify the process of cutting [5] a leather specimen. It was found that several toxic gases and substances were produced during leather processing, which may affect the operator’s health [6]. The physical–chemical analysis of leather during cutting can inform the waste elimination while cutting leather specimens [7]. The leather power wastage during the manufacturing of products has to be reduced as much as possible [8]. The developed product should be free from harmful and toxic substances [9]. The laser wastage can be eliminated using laser beam machining (LBM) process [10]. LBM is an unconventional thermal-process-assisted advanced machining that utilizes laser irradiation in the form of light to perform the operation. This machining technique makes use of a laser beam, which is a coherent high-power density light capable of cutting several metals or nonmetals. This approach uses laser radiation in the form of light to remove material from a workpiece surface through heating, melting, and vaporizing the material involved in the process. In this proposed method, diode laser cutting was applied to leather cutting [11]. This study aims to give extensive information on optimal power levels, cutting speeds and cut edge quality. CO_2_ laser cutting machines are commonly used to cut leather materials [12]. Semiconductor diode laser devices are extremely essential and have emerged as preferred instruments for a wide range of material processing applications due to their efficiency and affordable operating costs [13]. They are becoming more significant in industrial production processes, such as soldering, welding, hardening and cutting. Operational expenses can be drastically reduced with the use of diode lasers [14] for leather cutting. For diode lasers, maintenance is inexpensive and the predicted lifespan is long [15]. The major benefit of diode laser cutting over traditional laser cutting is the reduced optical power demand in relation to the workpiece thickness [16]. In the field of industrial laser cutting, it is widely recognized that the orientation of the laser beam has a substantial influence on the performance. There was a great deal of research on the linear and circular polarization states of carbon dioxide lasers [17]. The evolution of solid-state laser technology enabled the emergence of various optical approaches for polarization control of high-intensity laser beams [18]. It is vital to explore the influence of process parameters on response variables in order to enhance the efficiency of the laser beam machining process [19]. The process of interaction between the power diode laser and leather substrate involving carbonization was examined. The influence of laser cutting process parameters on cut quality was explored using this approach. Carbon particles produced during the burning of leather, which is a biomaterial, creates a layer along the cut in the carbonization zone. It is crucial to minimize this impact to maximize the product quality. It is essential to improve the health and safety of the operators and the environment throughout any machining operation. Laser power diodes minimize the carbonization impact on leather cutting because of their more controlled energy [20]. There is no standard technique available to quantify the carbonization on leather cutting [21].

From the detailed literature, only few studies have computed the impacts of carbonization on the surface of machined leather after the cutting process. No research has been available on the leather surface analysis using SEM and Fourier transform infra-red (FTIR). The use of laser diode technology in leather cutting has not yet been thoroughly investigated and examined. Hence, an endeavor was proposed using a machine vision system to study the effect of laser power diode on carbonization in leather. The carbon-related elements on the machined buffalo leather were identified using FTIR.

## 2. Experimental Methodology

**Figure 1:**
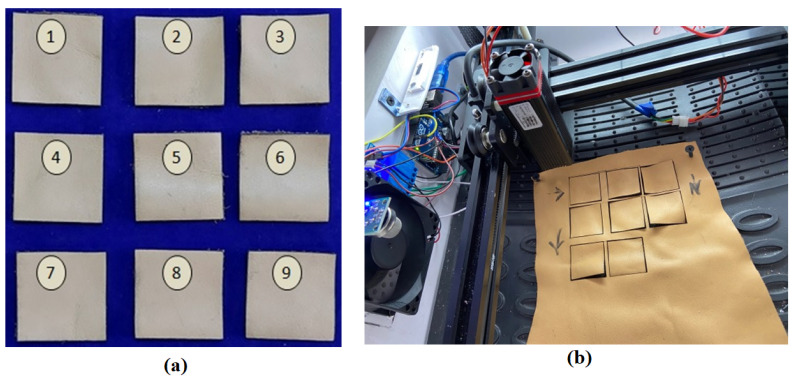
(**a**) Machined buffalo leather samples. (**b**) Leather cut performed using two-axis diode laser cutter.

Figure 1 shows a superior quality buffalo leather material that has better texture and can last much longer than the cow leather. This type of leather is used to manufacture rugged materials, such as shoes, wallets and bags, that last a lifetime. Buffalo leathers are around two to three times thicker than cow hide. In this investigation, a buffalo leather of 1.08 mm thickness and shade of light brown was used as a specimen because of its heavy usage. The leather material specimens have been provided by CSIR-Central Leather Research Institute, Chennai, India. The leathers were cut using 450 nm NEJE N30820 (Neje Laser tools, Guangdong, China) with an optical output power of 5.5 W. This blue diode laser of input power 20 W is used to cut the samples with size of 30 mm × 30 mm as shown in Figure 1. Surface quality is one of the most significant performance metrics in any machining operation.

**Figure 2:**
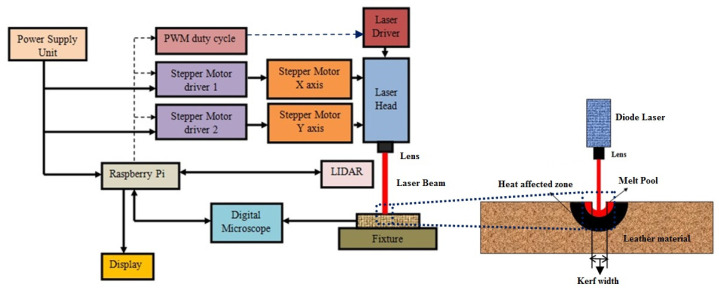
Functional block diagram of the proposed system.

The functional block diagram is represented in Figure 2. The power supply unit is capable of supplying power to various modules inside the system at the appropriate voltage. At maximum load, the Raspberry Pi 4 Model B with 1 GB RAM (Raspberry Pi Foundation, Cambridge, UK) needs a voltage of 5.1 V and a current of 2.5 A. Software installed on Raspberry Pi accepts the pattern of cut and information on the type of material. Depending on the feed rate specified by the controller, the stepper motor driver controls the stepper motors on both the x and y axes. The pulse width modulation (PWM) generated by the controller samples the input power according to the duty cycle. The sampled power is accepted by the laser driver that powers the laser module that finally makes the cut. The image acquired by the USB digital microscope is used to quantify the carbonization.

The Celestron 5MP CMOS imaging sensor handheld digital microscope (Manufactured by Celestron Instruments, Torrance, CA, USA) with 1600 × 1200 pixel array size was used to evaluate the surface quality. Micro-capture pro software was used to acquire the leather cross-section. To assess the surface quality, amplitude (voltage), duty cycle (percentage), and feed rate (mm/s) were chosen as input process parameters. The percentage of carbonization was determined by calculating the number of black and white pixels in the image obtained after the cut. The image was first converted to grayscale before being covered in black and white using a binary threshold technique. The frame-mounted diode laser has two axes, namely, x and y. Once the cut specification, including the pattern, was uploaded into the controller, the inputs were sent to the stepper motor, which drives the laser along the desired path. Through a regulated PWM signal, the intensity of the laser beam can be modified. The x-axis frame dimension is 20 mm × 20 mm, and it is kept perpendicular with the y-axis frame of 20 mm × 40 mm. The x-axis frame carries the diode laser module.

As shown in Table 1, the input process factors and their ranges were chosen. The amplitude is a power modulator that increases the power of diode laser. The frequency determines how often the signal is repeated over time. The frequency is proportional to the signal repetition. The material removal rate is influenced by the feed rate of the stepper motor. If the feed rate is low with optimized pulse duration, then the material removal rate will be significantly increased as the pulses will be introduced to the material in smaller increments. However, this parameter has to be optimized to suit the type of leather being machined as this also affects the heat-affected zone (HAZ).

Pulse width modulation (PWM) controls the average power supplied to the laser diode, thereby adjusting the output power. The duty cycle determines the pulse width of a signal. The duty cycle is determined as a percentage of the ratio of the pulse duration to the waveform total period. Table 2 displays various laser power diode cutting trials with quality measurements. The input and output parameters were preferred after considering inputs from various leather suppliers and laser machining specialists. The reason the output variable percentage of carbonization was considered as an output process parameter was because of its influence on productivity.

**Figure 3:**
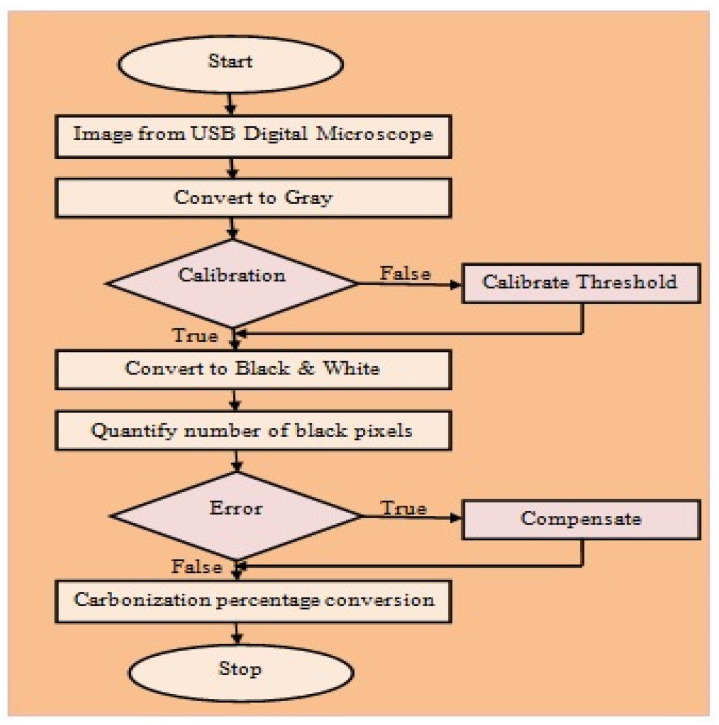
Flowchart for image acquisition.

The image obtained from the digital storage oscilloscope is converted to gray before any further image processing, as shown in Figure 3. The conversion of gray scale changes the colored image from a multi-dimensional to a more manageable two-dimensional figure. As leather is manufactured in a variety of shades, the calibration becomes a necessity for enhancing measurement accuracy. The leather cross-section captured using a digital microscope was analyzed to find out the equivalent gray intensity that is assigned as the threshold. The image is converted to black and white after calibration. This process converts all pixels with values above the threshold to black and every other pixel to white. The number of pixels was quantified and converted to a percentage after any necessary compensation of any error.

### 2.1. Measurement of Carbonization Percentage

When PWM is used, the power intensity of the diode laser can be varied by adjusting the duty cycle. The difficulties encountered while machining with diode lasers include dross and carbon layer formation. The objective of this research is to measure how much carbonization happens when leather is cut using a diode laser. The image-processing technique was used to quantify the amount of carbonization in the leather samples. An open-source python library called Open CV–Open computer vision was used to program the algorithm. This library includes all of the essential codes to achieve the desired outcome. The percentage of carbonization was estimated by using Equation (1).

(1) $$\mathrm{Carbonisation}\;\left(\%\right)=\frac{\mathrm{No}\;\mathrm{of}\;\mathrm{black}\;\mathrm{pixels}}{\mathrm{No}\;\mathrm{of}\;\mathrm{black}\;\mathrm{pixels}\;+\;\mathrm{No}\;\mathrm{of}\;\mathrm{white}\;\mathrm{pixels}\;}\times 100$$

Leather surface was examined using the FEI Quanta 200 Scanning Electron Micro-scope (manufactured by FEI Company, Hillsboro, OR, USA) under area mode. The utilization of the non-destructive method Fourier Transform Infra-Red (FTIR) spectroscopy (manufactured by IRTracer-100—Shimadzu, Kyoto, Japan) was to investigate the structural profiles of laser-machined leather samples throughout the stages of leather cutting. FTIR was used as a tool for the determination of organic compounds, including chemical bonds as well as organic content. The objective of this investigation was using the FTIR spectroscopy to access the chemical bond and structure of the material responsible for the carbonization layer in the machined buffalo leather after which the findings were compared to the literature. The image analysis is used to quantify the amount of carbonization (black substance) present in the work piece, while the FTIR result helps in analyzing the composition of this carbonization (black substance). The image analysis which revealed the amount of carbonization was further used to select the best combination of input parameters that could result in the least generation of this black substance. On the other hand, the FTIR analysis geared towards identifying its composition was used to understand the types of post-processing or filtration that can be used to clean the workpiece after cutting.

### 2.2. Design of Experiments and Optimization Approaches

The power intensity of the diode laser can be varied by adjusting the duty cycle. There are three input parameters, such as PWM amplitude, duty cycle, and feed rate. The L_9_ orthogonal matrix (OA) was chosen, as shown in Table 2, based on the Taguchi design, which can determine how many experiments should be executed. Carbonization was chosen as response parameter in the present study. Since the present study has dealt with a single response, i.e., carbonization only, the Taguchi methodology was used to obtain the optimal process parameters combination.

## 3. Results and Discussion

**Figure 4:**
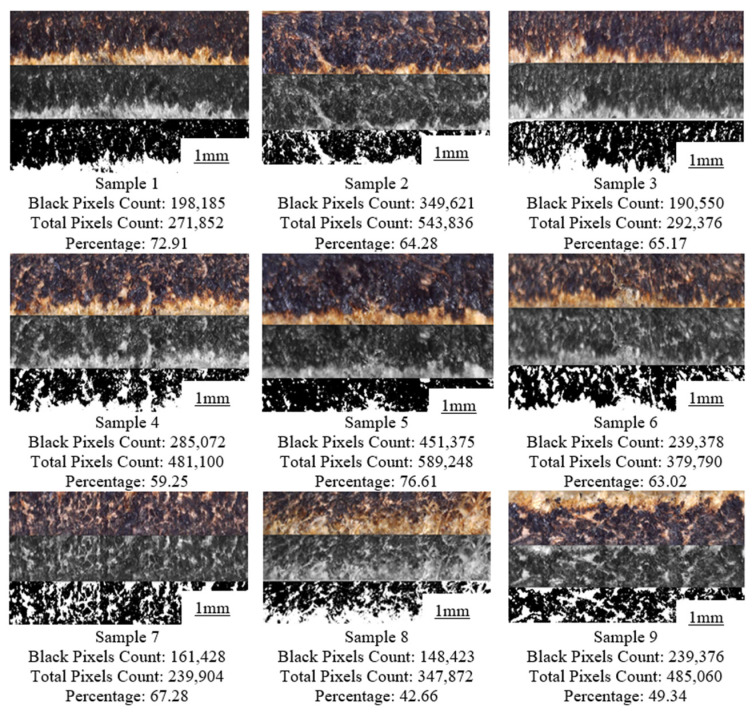
Conversion of captured image to binary image.

**Figure 5:**
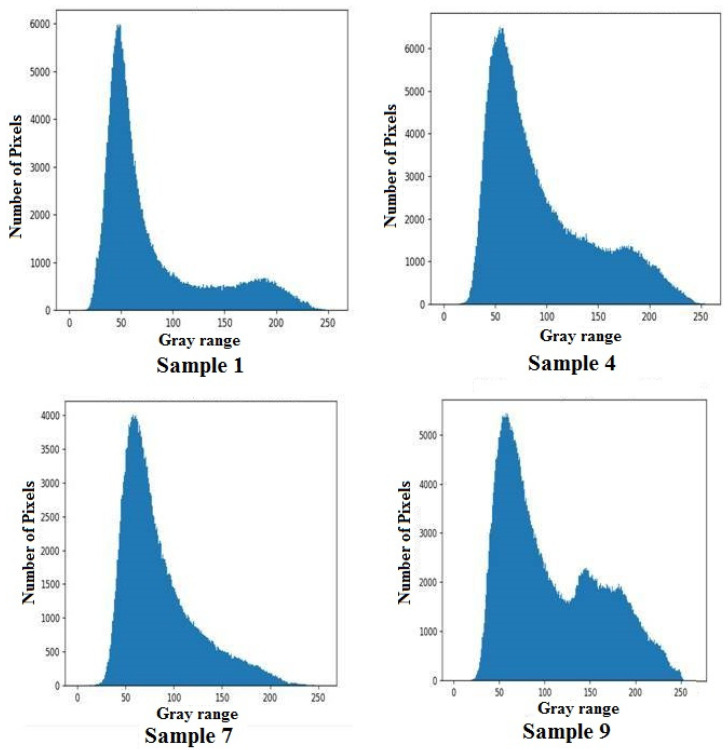
Histogram plot for buffalo leather samples.

Figure 4 illustrates the surface quality assessment of buffalo leather that was processed using a diode laser. The diode-based laser beam machining approach lowers the level of dross in the leather during the cutting operation. The machining method also revealed that no taper creation occurred. Nevertheless, an unwanted carbonization layer was noticed on the outermost surface of the machined surface. The distribution of radiant energy throughout the machining process affects the thickness and form of the carbonization layer. Figure 5 shows that Sample 5 has higher carbonization with a higher black pixel count out of the total pixel count. Sample 8 produces better surface quality with the least carbonization.

### 3.1. Histogram Plot Analysis

Histogram is a plot with gray pixel range in the x-axis and the number of pixels in the image on the y-axis. The pixel count on the gray image for Samples 1, 4,7 and 9 are as shown in Figure 5. An image histogram depicts the distribution of pixel intensity for a gray scale image in a digital picture graphically. In the present study, a histogram plot is used to depict the distribution of carbonization following the machining process since it is not equally distributed throughout the leather. Zero represents a black pixel and 255 represents a white pixel in the gray scale. The carbonization due to diode laser cutting can be seen in the laser-machined leather. This creates a larger number of pixels with lower values on the gray scale, which is represented by a higher y-axis value on the left side of the x-axis. Furthermore the brighter region in the image creates more bars at higher values on the x-axis, indicating the amount of non-carbonized region.

### 3.2. Surface Texture Analysis

**Figure 6:**
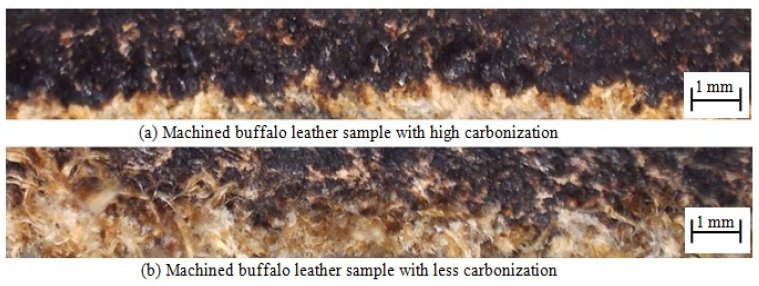
Carbonization layer on laser-machined leather under trial 5.

The carbonization layers were formed due to diode laser cutting under Trial 5 as depicted in Figure 6. Though the diode laser cutting operation was carried out with a minimal output power of 5.5 W, a substantial quantity of carbonization was noticed on the machined layer. Through visual observation, it was noted that the impact of diode laser cutting process on carbonization zone in machined buffalo leather is higher in Figure 6a than in Figure 6b.

### 3.3. Scanning Electron Microscopy (SEM) with EDX for Elemental Analysis

**Figure 7:**
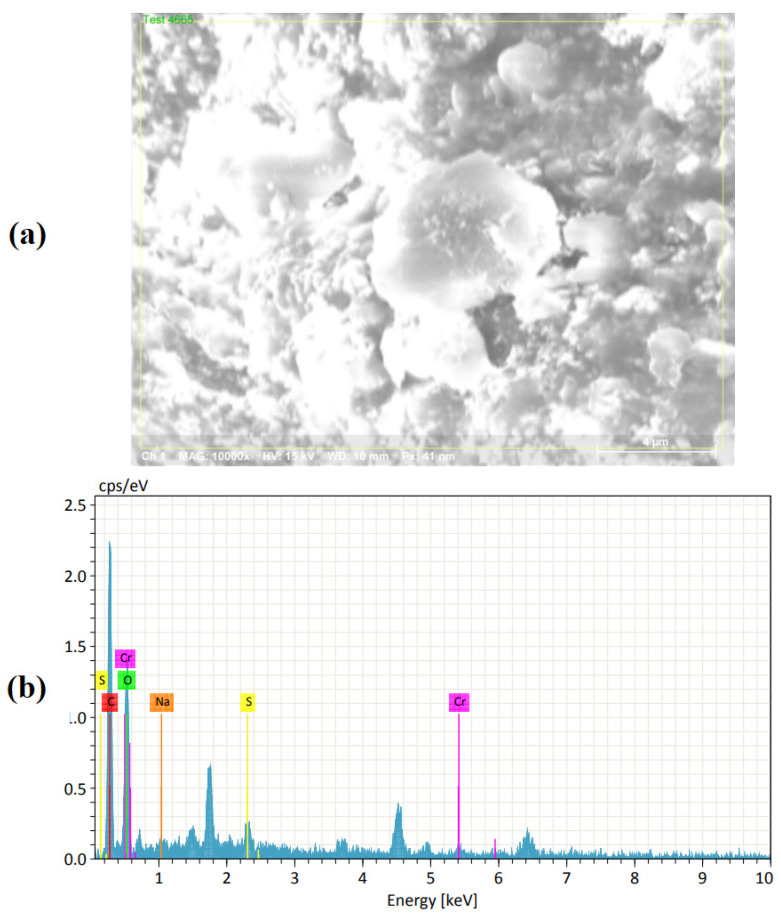
Deteriorated machined buffalo leather using (**a**) FEI Quanta 200 Scanning Electron Microscope (**b**) EDX for elemental analysis.

The leather surface was examined using the FEI Quanta 200 Scanning Electron Microscope under area mode, as shown in Figure 7. This was used to investigate the changes in the surface morphology. The EDX was used for elemental analysis characterization. There was a total deformation of the surface morphology due to carbonization. According to EDX analysis shown in Table 3, laser-machined leather utilized a large amount of carbon in the buffalo leather (50.93%), while oxygen rated 45.02%. The amount of sulfur, chromium and sodium found in machined buffalo leather with a range of 0.1% to 2.1%.

### 3.4. Spectrum in the FTIR Analysis Report

**Figure 8:**
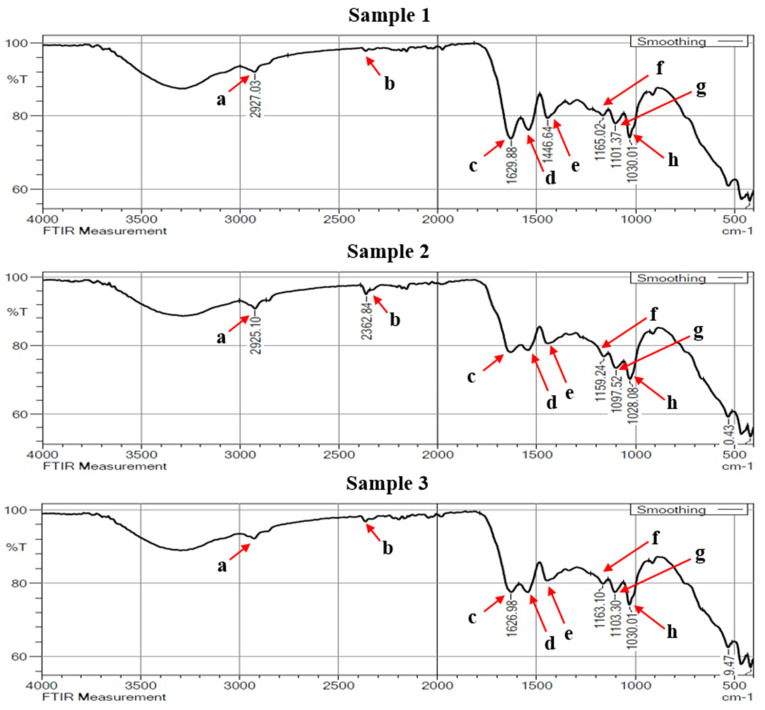
FTIR spectra of buffalo leather samples 1 to 3.

**Figure 9:**
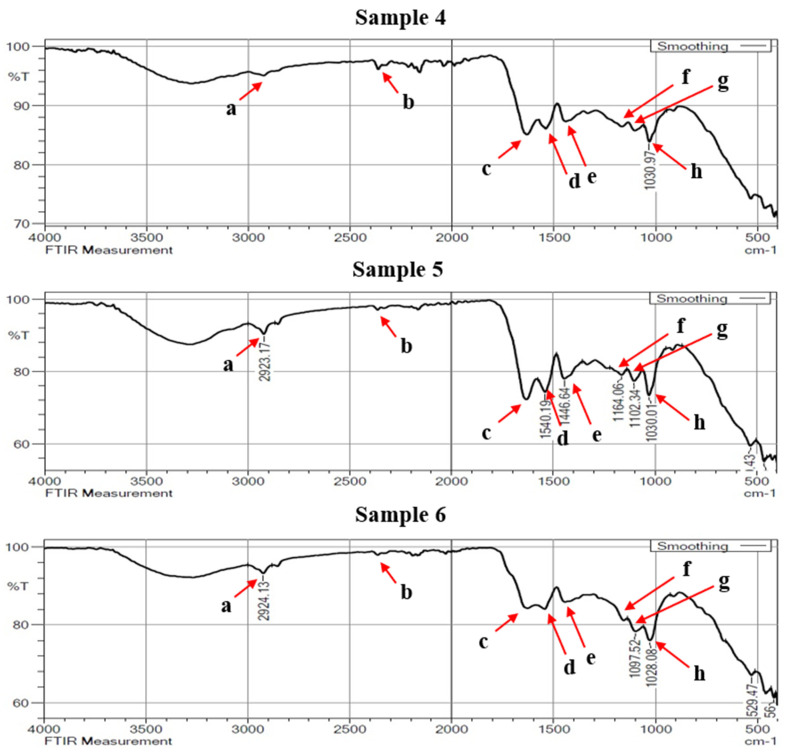
FTIR spectra of buffalo leather samples 4 to 6.

**Figure 10:**
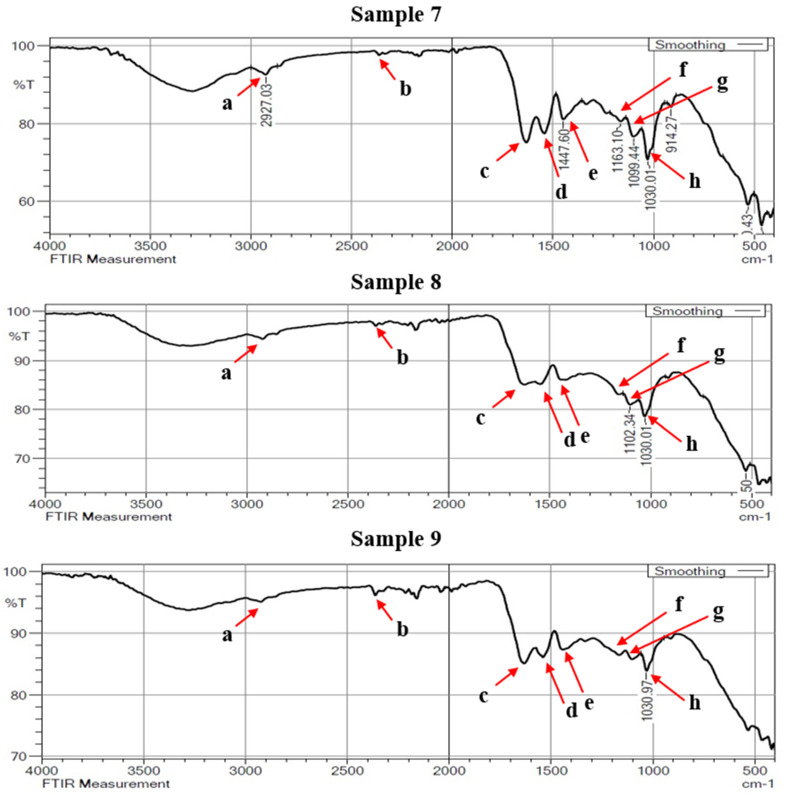
FTIR spectra of buffalo leather samples 7 to 9.

Figure 8, Figure 9 and Figure 10 showed the enlarged FTIR spectra at different regions, which range from 400 to 4000 cm^−1^. The specific frequency of each functional group is available in Table 4. From the double-bond region (1500–2000 cm^−1^) it was observed that the presence of carbonyl compound (C=C) was at the spectral frequency of (1850–1650 cm^−1^). There is only one peak of absorption revealing the hydrogen bond in identifying the single-bond region (2500–4000 cm^−1^). It is also essential to analyze absorption below 3000 cm^−1^ when diagnosing unsaturated bonds.

The absorption identified in the range of 2923 and 2927 cm^−1^ is intended for C-H stretching mode in the carbonyl group. A strong peak at 2362 cm^−1^ observed in the triple-bond region indicates the absorption band of C≡C. This peak is usually followed by the occurrence of some more additional spectra at the frequencies of 1600–1300 cm^−1^ and 1200–1000 cm^−1^. In the double-bond region, a peak between 1626 cm^−1^ and 1629 cm^−1^ shows the carbonyl (C=C), indicating the existence of carbonyl compound carboxyl. Carbon with a double bond has an unsaturation band nearer to 1650 cm^−1^. With intense or strong absorption bonds, typical conjugations with a C=C structure diminish the intensity frequency.

The presence of carboxylate for consistent and rapid acidification of the pelts is shown by the usual absorption band with a wave number of 1540 cm^−1^. The aromatic ring stretching and vibration (C=C-C) in lignin was assigned by the IR absorption band at 1446 cm^−1^. The lignin is used to strengthen the stiffness, thickness and firmness of the leather. The spectra recorded in 1663 cm^−1^ indicate the CO stretching. This could be due to the possible nutrient type protein and collagen. Peaks determining during 1097 cm^−1^ and 1028 cm^−1^ are the characteristics of C-O stretch.

### 3.5. Optimization of the Process Parameters to Reduce the Carbon Layer Formation

Since the present study has dealt with single response, i.e., carbonization only, the Taguchi methodology was used to obtain the optimal process parameters combination. It is essential to reduce the carbon layer formation for increasing the product quality and reducing the adverse environmental effects. Table 5 displays carbon layer quality measurements, which can be derived from Figure 5. All trials were conducted three times and the mean value was considered as the final value. The computed standard deviation values were also found as an acceptable range as depicted in Table 5. It was found that Sample number 8 could produce less carbonization region among the conducted trials. The higher amplitude of the voltage signal with lower feed rate could effectively remove the material, and thus, produce efficient cuts. Table 6 shows the optimal combination of process to reduce the carbonization region. The values were computed based on the average response across all the levels of the factors. The carbon layer, which is formed based on thermal energy and its distribution over time, depends on the amplitude of the signal and its distribution can be influenced by the duty cycle and feed rate. While the lower duty cycle with a higher feed rate can reduce distribution over the time, a lower amplitude can reduce the thermal energy. Hence, these optimal factors can reduce the carbon formation for enhancing a positive environmental impact on leather cutting. The highest max min value indicated higher significance of input parameters on the carbonization region. Since the amplitude could affect the laser power intensity, it could possess higher influential on determining the carbonization region.

## 4. Conclusions

The impact of a laser power diode on carbonization in leather cutting was investigated using FTIR analysis in an experimental study. The following conclusions were drawn from the experimental analysis.

➢ Carbonization can be measured directly using proposed image processing in the leather industries;
➢ The lower amplitude duty cycle with higher feed rate can reduce carbon formation owing to the lower thermal energy distribution;
➢ The digital microscope-based machine vision system and the FTIR spectrum can quantify the carbonization using open-source python library;
➢ The amplitude (4.5 V), duty cycle (70%) and feed rate (90 mm/s) can produce optimal performance measures. Since the amplitude could affect the laser power intensity, it can possess higher influential on determining the carbonization region.

## Figures and Tables

**Table 1:** Process variables.

Sl. No	Parameters	Units	Level 1	Level 2	Level 3
1.	Amplitude	V	4.50	4.75	5.0
2.	Duty cycle	%	70	80	90
3.	Feed rate	mm/s	0.803	0.889	1.185

**Table 2:** Experimental trial in the proposed study.

Experimental Trial	Amplitude (V)	Duty Cycle (%)	Feed Rate (mm/s)
1	4.5	70	0.803
2	4.5	80	0.889
3	4.5	90	1.185
4	4.75	70	0.889
5	4.75	80	1.185
6	4.75	90	0.803
7	5	70	1.185
8	5	80	0.803
9	5	90	0.889

**Table 3:** Elemental analysis of laser-machined leather.

Samples	Chemical Composition (Atomic %)				
Machined Buffalo Leather	C	O	S	Cr	Na
	50.93	45.02	2.10	1.79	0.17

**Table 4:** Functional groups and their quantified frequencies.

Assignment	Wave Numbers (cm^−1^)		Functional Group
	From Literature	From Experiment	
a	2923–2930	2923–2927	C-H stretching
b	2350	2362	C≡C (triple bond)
c	1670–1620	1626–1629	C=C (Double bond)
d	1610–1550	1540	Carboxylate
e	1510–1450	1446	C=C-C Aromatic ring stretch
f	1160	1163	C-O stretching
g	1100	1097	-C-O (eter) stretching
h	1030	1028	-C-O (eter) stretching

**Table 5:** Experimental outcome.

Sample Number	Carbonization Region (%)	Standard Deviation (%)
1	72.91	2.65
2	64.28	2.21
3	65.17	2.26
4	59.25	1.96
5	76.61	2.83
6	63.02	2.15
7	67.28	2.36
8	42.66	1.13
9	49.34	1.47

**Table 6:** Optimal combination of factors.

Factor	Level 1	Level 2	Level 3	Max Min	Optimal Factors
Amplitude	67.45	66.29	53.09	14.36	4.5 V
Duty cycle	66.48	61.18	59.18	7.30	70%
Feed rate	59.53	57.62	69.69	12.07	90 mm/s

## Data Availability

Not applicable.
